# Engineering a mouse-adapted SADS-CoV and establishing a neonatal mouse model to study its infection

**DOI:** 10.1128/mbio.03246-25

**Published:** 2026-01-12

**Authors:** Hanyu Zhang, Mengdi Zhang, Jiaru Zhou, Pengfei Li, Ran Jing, Hongmei Zhu, Yifei Lang, Qigai He, Mengjia Zhang, Wentao Li

**Affiliations:** 1 National Key Laboratory of Agricultural Microbiology and Hubei Hongshan Laboratory, Huazhong Agricultural University 47895, Wuhan, Hubei Province, China; 2 The Cooperative Innovation Center for Sustainable Pig Production, Huazhong Agricultural University 47895, Wuhan, Hubei Province, China; 3 College of Veterinary Medicine, Huazhong Agricultural University 627716, Wuhan, Hubei Province, China; 4 College of Veterinary Medicine, Sichuan Agricultural University 12529, Chengdu, Sichuan Province, China; 5 Frontiers Science Center for Animal Breeding and Sustainable Production, Wuhan, Hubei Province, China; 6 Hubei Jiangxia Laboratory, Wuhan, China; Ulm University Medical Center, Ulm, Germany

**Keywords:** swine acute diarrhea syndrome coronavirus, mouse hepatitis virus, mouse infection model, antiviral drugs

## Abstract

Swine acute diarrhea syndrome coronavirus (SADS-CoV) is an emerging bat-origin alphacoronavirus causing severe disease in neonatal piglets, with significant economic losses to the swine industry. The virus exhibits a broad species tropism, infecting cells derived from pigs, humans, and mice, highlighting its potential for cross-species transmission. Due to drawbacks associated with the use of young piglets, there is a need for an appropriate small animal model to study SADS-CoV biology. Here we established a mouse infection model based on a murinized mutant of the virus, mSADS-CoV, in which the ectodomain of the viral spike protein was replaced by that of the murine coronavirus mouse hepatitis virus. This chimeric virus, generated through targeted RNA recombination, replicated efficiently in murine cell cultures and exhibited an age-dependent infection in neonatal mice that was lethal in 2-day-old BALB/c mice, affecting various organs, notably the intestine. We validated our infection model by successfully verifying the efficacy of the RNA-dependent RNA polymerase inhibitor remdesivir. The model will serve as a valuable tool for studying SADS-CoV pathogenesis and for elucidating the roles of host factors in viral replication as well as for preclinical evaluation of antiviral compounds targeting the viral replication machinery.

**IMPORTANCE:**

Swine acute diarrhea syndrome coronavirus (SADS-CoV) poses a threat to the swine industry and public health because of its broad species tropism and potential for cross-species transmission. The emergence of other bat-derived coronaviruses, including severe acute respiratory syndrome coronavirus (SARS-CoV), SARS-CoV-2, and Middle East respiratory syndrome coronavirus, underscores the need for robust models to study these pathogens. The successful rescue of mSADS-CoV and the development of a mouse infection model represent significant advancements in SADS-CoV research. This model not only enables the evaluation of antiviral therapeutics such as remdesivir but also provides a powerful platform for investigating viral replication mechanisms and host–pathogen interactions, offering critical insights for pandemic preparedness.

## INTRODUCTION

Swine acute diarrhea syndrome coronavirus (SADS-CoV) is a recently identified porcine enteric coronavirus that predominantly affects neonatal piglets. Clinically, SADS-CoV presents symptoms strikingly similar to those caused by other known porcine enteric coronaviruses, including acute diarrhea and vomiting (1, 2). Since its initial identification in Guangdong, China, in 2017, SADS-CoV has re-emerged in multiple regions, including Guangdong, Fujian, and Guangxi, giving rise to significant economic losses to the swine industry (3–5). Emerging evidence suggests that SADS-CoV is likely of bat origin (2). Notably, other bat-derived coronaviruses, such as SARS-CoV-2, severe acute respiratory syndrome coronavirus (SARS-CoV), and Middle East respiratory syndrome coronavirus (MERS-CoV), have previously caused global outbreaks, posing severe threats to public health and the global economy (6–8). This raises concerns that SADS-CoV may also hold a risk to human health through infection of an intermediate host. Studies have demonstrated that SADS-CoV can infect human cell lines, including Huh7.5, Caco-2, and ST-INT, and can efficiently replicate in cells derived from various other species, such as pigs, chickens, and mice, highlighting its significant potential for cross-species transmission (9, 10). To date, no effective therapeutic agents or vaccines have been developed for the prevention or treatment of SADS-CoV, underscoring the critical importance of research into its pathogenic mechanisms.

While porcine neonates serve as natural reservoirs for SADS-CoV, the substantial experimental costs, inconsistent reproducibility, and technical complexity associated with porcine infection models significantly limit their utility in laboratory investigations. Mouse models generally offer distinct advantages for experimentation.

However, the few SADS-CoV infection studies in mice reported so far were inconclusive. Oral or intraperitoneal inoculation of 6- to 8-week-old immunocompetent C57BL/6J mice only caused subclinical infection with replication, particularly in the spleen (10). In contrast, similar inoculations of 10-week-old immunocompetent BALB/c and immunodeficient IFNR mice failed to induce detectable replication (9). Age-dependent susceptibility was observed in both BALB/c and C57BL/6J mice upon intragastric inoculation: neonates (<7 days old) showed the highest vulnerability, which gradually declined with age; animals 3 to 4 weeks and older appeared resistant (11). Successful infection was also observed after intracranial inoculation in 7- and 14-day-old BALB/c mice (12). However, as a mouse model for antiviral research, the intracranial route of infection appears suboptimal since it poses a major constraint, given the poor blood–brain barrier penetration of most clinical antiviral compounds. Altogether, these various findings emphasize the critical need for more optimized mouse infection models.

SADS-CoV is an enveloped, positive-sense RNA virus distinguished by typical surface spike glycoprotein protrusions. The 27.2 kb genome encodes four principal structural proteins: the spike glycoprotein and the envelope (E), membrane (M), and nucleocapsid (N) proteins (2). Proteolytic processing divides the spike glycoprotein into S1 and S2 subunits, with the S1 subunit mediating receptor recognition and binding, while host proteases critically regulate S2 subunit activation to facilitate viral-host membrane fusion, a mechanism evolutionarily conserved among coronaviruses (13). This functional specialization establishes the S1 subunit as the critical determinant of viral tissue tropism and host range (14, 15). Owing to the essential biological role of the S glycoprotein, targeted RNA recombination strategies predominantly utilize S gene replacement to achieve viral recombination and selection (16).

Capitalizing on this molecular feature, we engineered in the present study a chimeric recombinant SADS-CoV carrying mouse hepatitis virus (MHV) spike protein through targeted RNA recombination, designated as mSADS-CoV. Upon intraperitoneal administration in neonatal BALB/c mice (2 days old), mSADS-CoV demonstrated robust replication competence with subsequent production of infectious virions. To test the performance of our animal model, we evaluated the efficacy of the broad-spectrum antiviral agent remdesivir (RDV) against mSADS-CoV infection. The results demonstrated that RDV effectively suppressed viral replication *in vivo* and mitigated pathological tissue damage in infected mice. Notably, the model confirmed the physiological expression of replicase-associated genes during mSADS-CoV infection. The established mSADS-CoV infection system provides a valuable platform for various purposes, including screening of replication-targeting antiviral agents and functional validation of host factors mediating coronaviral replication.

## RESULTS

### Rescue of mSADS-CoV via targeted RNA recombination technology

**Fig 1:**
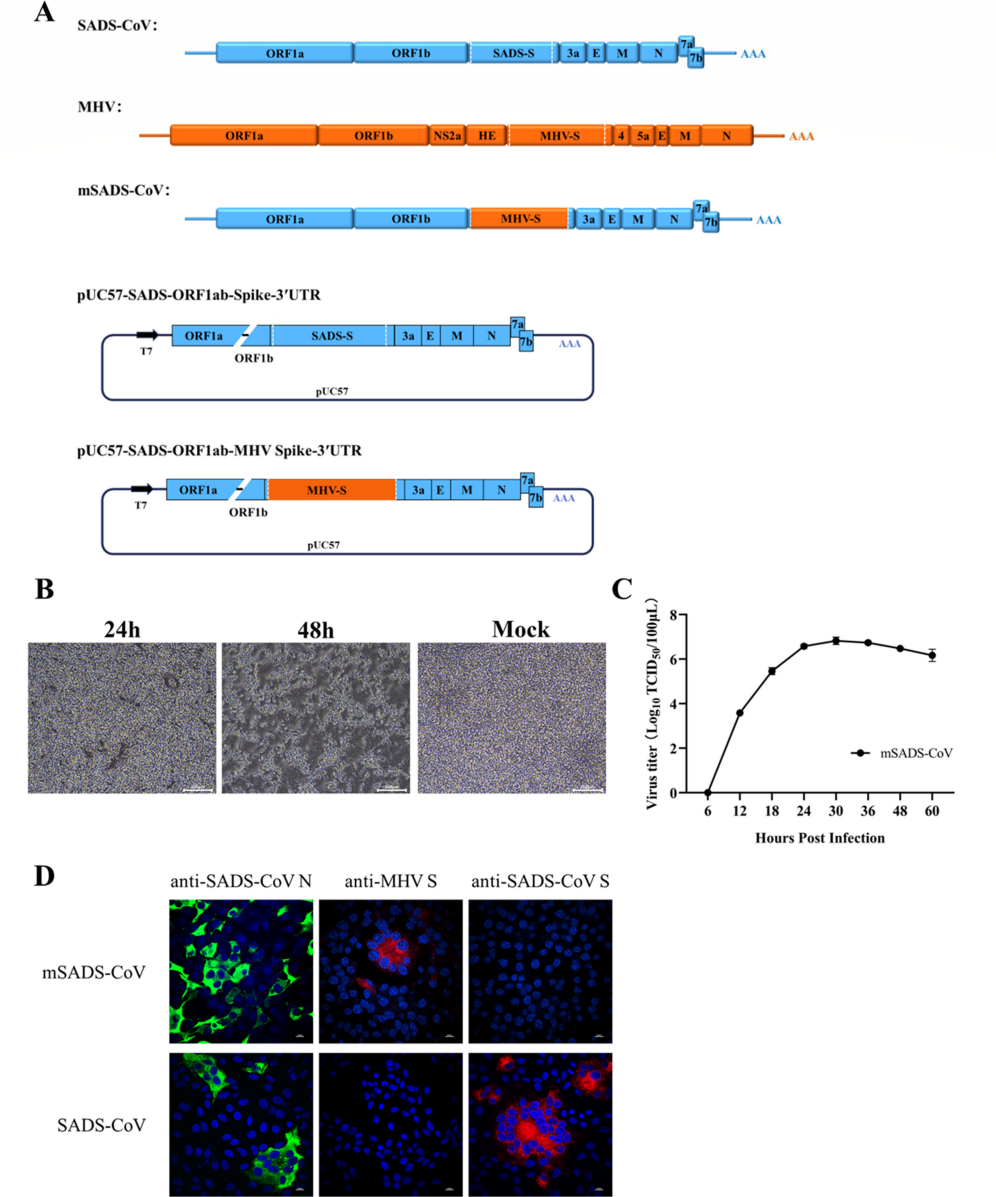
Rescue and validation of the recombinant virus mSADS-CoV. (**A**) Schematic representation of the generation of mSADS-CoV by homologous recombination of RNA, transcribed *in vitro* from pUC57-SADS-ORF1ab-MHV Spike-3′UTR and transfected into SADS-CoV-infected Vero cells. (**B**) Cytopathic effects of recombinant mSADS-CoV in LR7 cells 24 and 48 h post-infection. (**C**) Growth kinetics of mSADS-CoV in LR7 cells. Cells were infected with mSADS-CoV at an MOI of 0.01, and viral titers were determined by TCID_50_. (**D**) IFA of LR7 cells infected with mSADS-CoV using an anti-SADS-CoV S monoclonal antibody, an anti-MHV S protein antibody, and an anti-SADS-CoV N protein antibody. Vero cells infected with SADS-CoV were used as a control. Error bars represent the standard deviation.

With the aim of developing a murine animal model for SADS-CoV, we engineered a murinized mutant of the virus, mSADS-CoV, by replacing the ectodomain of the spike protein by that of MHV using the prototype targeted RNA recombination technology (17) (Fig. 1A). The first step in the construction process involved the generation of a so-called transfer vector consisting of a genomic SADS-CoV cDNA from which ORF1a and almost all ORF1b are lacking. The small 5′-terminal genomic fragment and the ORF1b-Spike-3′UTR fragment were amplified from the SADS-CoV cDNA template and ligated, meanwhile introducing a T7 promoter at the 5′-terminus. The amplified fragments were subsequently cloned into the pUC57 plasmid vector, generating pUC57-SADS-ORF1ab-Spike-3′UTR (Fig. 1A). The proper spike gene sequence within this plasmid was then replaced by the corresponding MHV spike gene sequence amplified from MHV genomic cDNA, resulting in the plasmid pUC57-SADS-ORF1ab-MHV Spike-3′UTR (Fig. 1A). This plasmid was linearized and *in vitro* transcribed using T7 RNA polymerase, and the resultant mRNA was electroporated into SADS-CoV-infected Vero cells, which were subsequently overlaid onto mouse LR7 cells. Distinct cytopathic effects (CPEs) were observed in the LR7 cells (Fig. 1B), indicating that recombinant virus mSADS-CoV had been generated. The virus was purified by two rounds of plaque purification, after which its genomic structure was verified by sequencing. It grew in LR7 cells to a titer exceeding 10^6^ 50% tissue culture infective doses (TCID_50_)/mL, its replication kinetics showing a typical growth pattern (Fig. 1C). The identity of mSADS-CoV was further confirmed through indirect immunofluorescence assay (IFA), wherein all infected cells exhibited positive reactivity to both anti-MHV spike protein antibodies and anti-SADS-CoV N protein antibodies (Fig. 1D), thereby validating the chimeric virus’s purity and structural identity.

### Infection of two mouse strains with mSADS-CoV

**Fig 2:**
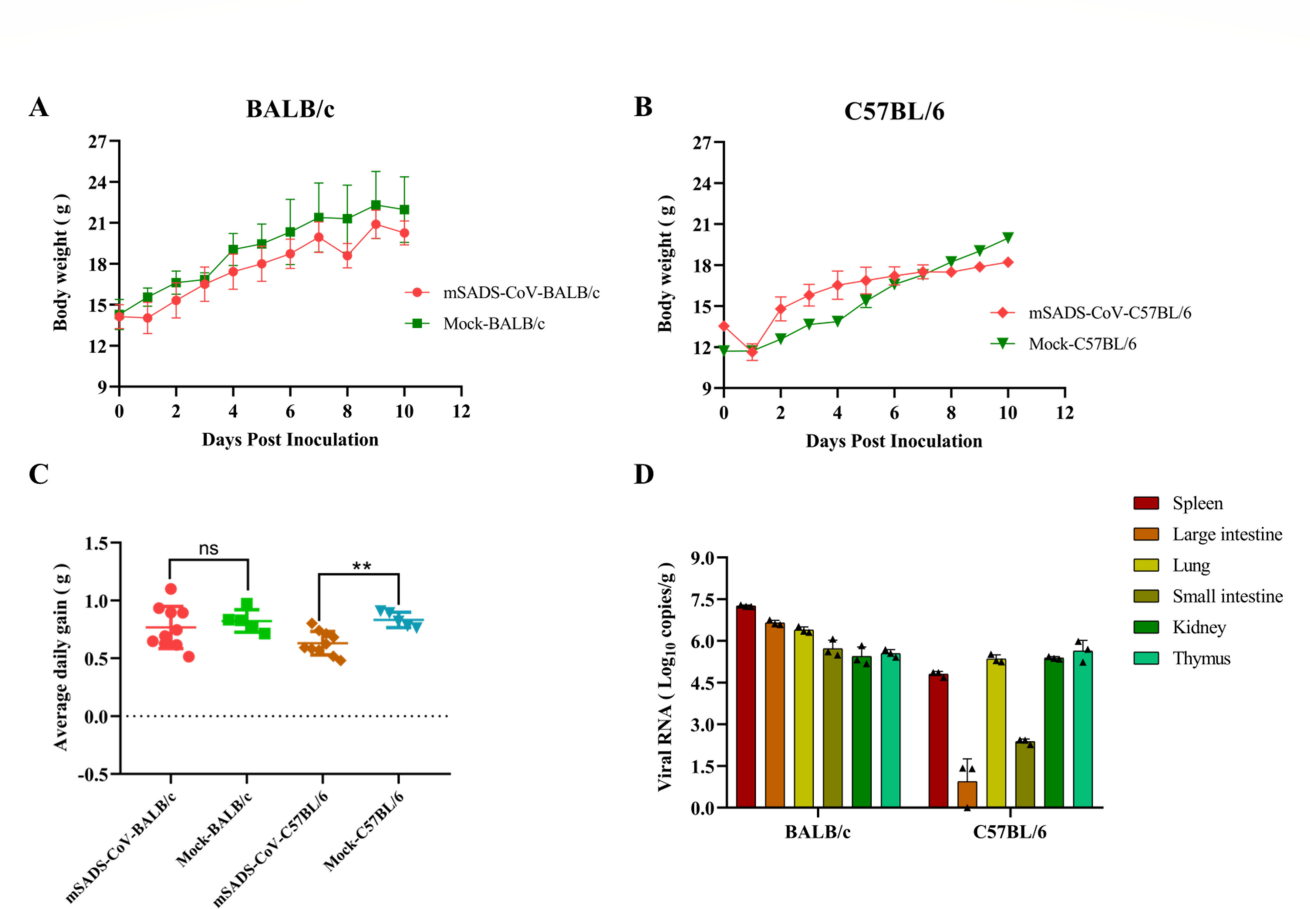
Infection of 3-week-old BALB/c and C57BL/6 mice with mSADS-CoV. (**A**) Body weight changes in BALB/c mice following the intraperitoneal administration of mSADS-CoV. (**B**) Body weight changes in C57BL/6 mice following the intraperitoneal administration of mSADS-CoV. (**C**) Average daily weight gain of BALB/c and C57BL/6 mice inoculated with mSADS-CoV via i.p. injection. (**D**) Viral loads in the spleen, large intestine, lung, small intestine, kidney, and thymus of BALB/c and C57BL/6 mice at 5 days post-inoculation by quantitative reverse transcription polymerase chain reaction. Each data point represents the mean of three technical replicates per sample. Error bars represent the standard deviation. **, *P* < 0.01; ns means not significant.

To investigate the susceptibility of mice to mSADS-CoV, the virus was inoculated via intraperitoneal injection into young, 3-week-old mice from two different strains, BALB/c and C57BL/6. The clinical symptoms and body weight changes in the animals were monitored daily. Mice from neither group developed obvious clinical symptoms or mortalities. In BALB/c mice, no significant difference in average daily weight gain between the infected and control groups was observed; mSADS-CoV infection did not affect the animals’ weight gain (Fig. 2A and C). In C57BL/6 mice, an initially more rapid increase in body weight in the infected animals relative to the controls was soon followed, from 3 to 4 days post-inoculation, by a clear growth retardation (Fig. 2B), which appeared to be significant as judged by a comparison of the average daily weight gain in each group (Fig. 2C).

Tissue samples from the mice were subjected to quantitative reverse transcription polymerase chain reaction (qRT-PCR). Viral loads in these tissues as measured at 5 days post-infection (dpi) are shown in Fig. 2D. Except for the thymus, the viral loads in positive tissues of BALB/c mice were higher than those in C57BL/6 mice, with the viral loads particularly in intestines and lungs of C57BL/6 mice being significantly lower than those in other tissues. These results indicate that both BALB/c and C57BL/6 mice can be infected by mSADS-CoV through intraperitoneal injection, but that both strains exhibit subclinical infection. Based on the viral loads observed in the various organs, the virus replicates to higher titers in BALB/c compared to C57BL/6 mice.

### Infection of 2- and 7-day-old mice with mSADS-CoV

**Fig 3:**
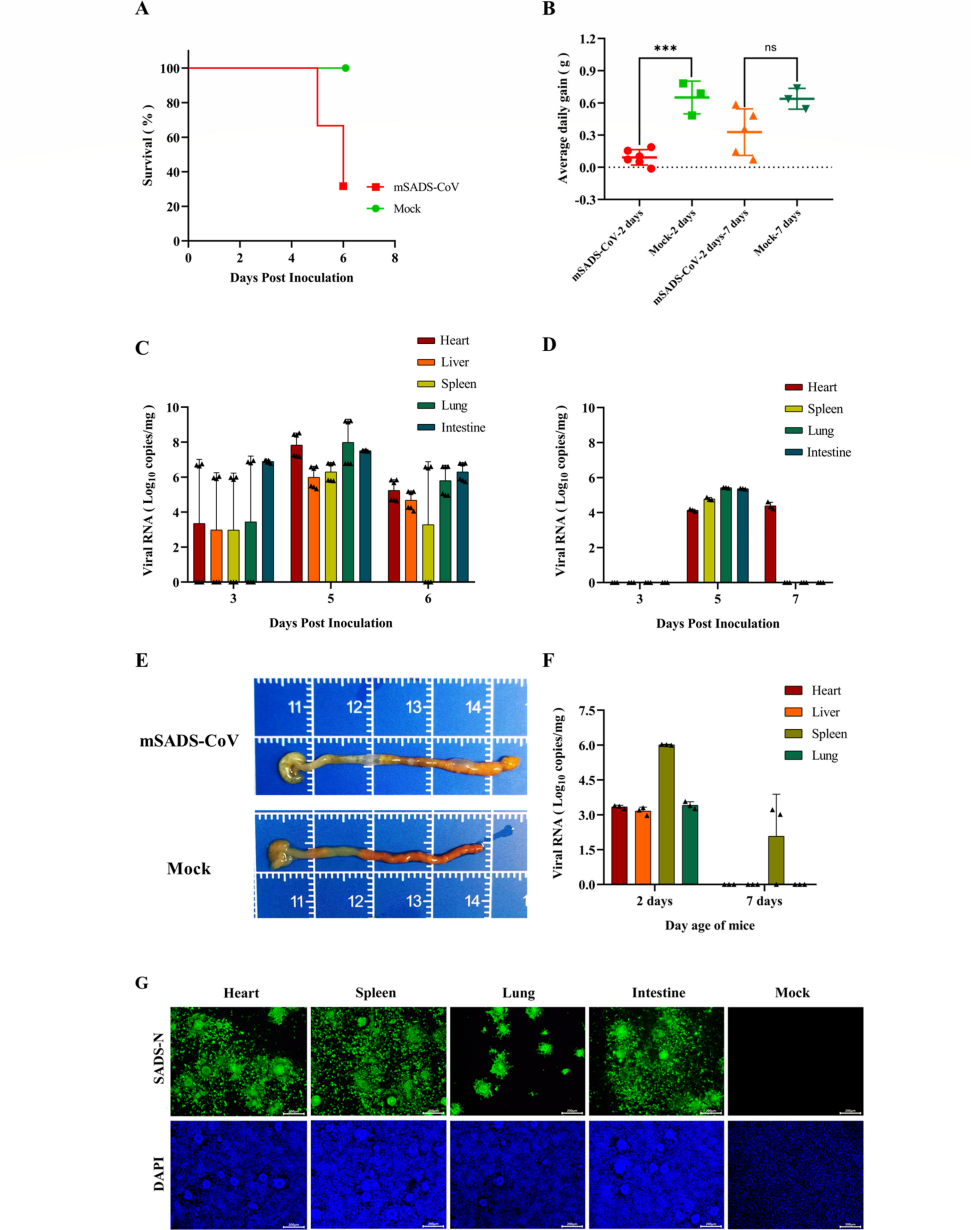
Infection of 2- and 7-day-old BALB/c mice with mSADS-CoV. (**A**) Survival curve of 2-day-old BALB/c mice following i.p. injection of mSADS-CoV. (**B**) Average daily weight gain in 2-day-old versus 7-day-old BALB/c mice following i.p. injection of mSADS-CoV. (**C**) qRT-PCR determination of viral loads in the heart, liver, spleen, lung, and intestine of 2-day-old BALB/c mice after i.p. mSADS-CoV infection. Each data point represents an individual technical replicate. (**D**) qRT-PCR determination of viral loads in the heart, liver, spleen, lung, and intestine of 7-day-old BALB/c mice after i.p. mSADS-CoV infection. Each data point represents an individual technical replicate. (**E**) Pathological alterations in the large intestine of 2-day-old BALB/c mice at 5 dpi with mSADS-CoV. (**F**) Viral loads in the heart, liver, spleen, and lung of 2- and 7-day-old BALB/c mice at 3 dpi with SADS-CoV, as measured by qRT-PCR. The intestine tested negative in all cases. Each data point represents an individual technical replicate. (**G**) IFA confirmation of successful isolation of tissue-derived virus using anti-SADS-CoV N protein antibody. Error bars represent the standard deviation. ***, *P* < 0.001; ns means not significant.

In our search for a clinically more representative mouse model, we conducted mSADS-CoV infection experiments using 2- and 7-day-old BALB/c mice, taking SADS-CoV along in parallel for comparison. Inoculation of 2-day-old mice with mSADS-CoV resulted in the development of severe infection and disease. All animals died at 5 or 6 dpi or had to be euthanized at 6 dpi (Fig. 3A). Some animals were already euthanized for necropsy at 3 dpi. In contrast, in the 7-day-old challenge group, no obvious disease was observed, and all mice survived. Body weight monitoring revealed that mSADS-CoV infection significantly inhibited normal weight gain in 2-day-old infected mice, whereas the average daily weight gain in the 7-day-old challenge group was not significantly different from that in the control group (Fig. 3B). Quantitation of viral RNA by qRT-PCR in several organs revealed that in the 2-day-old challenge group, heart, liver, spleen, lungs, and intestines were all positive, reaching the highest titers of around 10^8.0^ copies/mg in the lungs and hearts at 5 dpi (Fig. 3C). In the 7-day-old challenge group, only the heart, lungs, spleen, and intestines at 5 dpi and the heart at 7 dpi were positive, the highest titers (in the lungs) being only 10^5.4^ copies/mg (Fig. 3D). Necropsy of the mice from the 2-day-old challenge group at 5 dpi revealed that the walls of their large intestine were thinned, swollen, and contained foamy contents (Fig. 3E). To verify the presence of infectious viral particles in the positive tissues of the mice, tissue homogenates were prepared, and their supernatants were inoculated onto LR7 cells. As shown by the IFA results (Fig. 3G), all tested tissues appeared to contain infectious virus. These results indicate that mSADS-CoV can effectively replicate in various tissues of 2-day-old BALB/c mice, inhibiting weight gain and causing mortality, but that its infection efficacy decreases rapidly with age as no obvious clinical disease was observed when the animals were infected when 7 days old.

Intraperitoneal injection of BALB/c mice of the same ages and with the same dose of SADS-CoV did not elicit any significant clinical symptoms or weight loss. Yet, the animals had become infected as shown by the results of qRT-PCR analysis on some tissues collected at 3 dpi. In the 2-day-old challenge group, positive tissues included the heart, liver, kidney, and, in particular, the spleen, whereas in the 7-day-old challenge group, only the spleen was positive, though with significantly lower titer (Fig. 3F). These findings indicate that SADS-CoV can replicate—though poorly—in BALB/c mice, its infection exhibiting similar characteristics as mSADS-CoV, in particular with respect to their similar host age dependence. In both cases, the virus is cleared quite rapidly by the host, making sustained infection difficult. We conclude that mSADS-CoV infection of 2-day-old BALB/c mice offers a suitable mouse infection model for SADS-CoV.

### Testing the mSADS-CoV mouse model by evaluating the antiviral efficacy of RDV

To test the applicability of the mSADS-CoV mouse infection model, we selected the broad-spectrum antiviral drug RDV for *in vivo* antiviral efficacy evaluation. We first checked whether RDV can inhibit mSADS-CoV infection *in vitro*. Thus, parallel cultures of LR7 cells were incubated for 2 h with maintenance medium containing different concentrations of RDV. The culture supernatants were then replaced with culture media containing the same RDV concentrations and 0.1 MOI mSADS-CoV and incubated for another 2 h. The cells were then washed twice with phosphate-buffered saline (PBS) and incubated with maintenance medium containing again the same concentrations of RDV.

**Fig 4:**
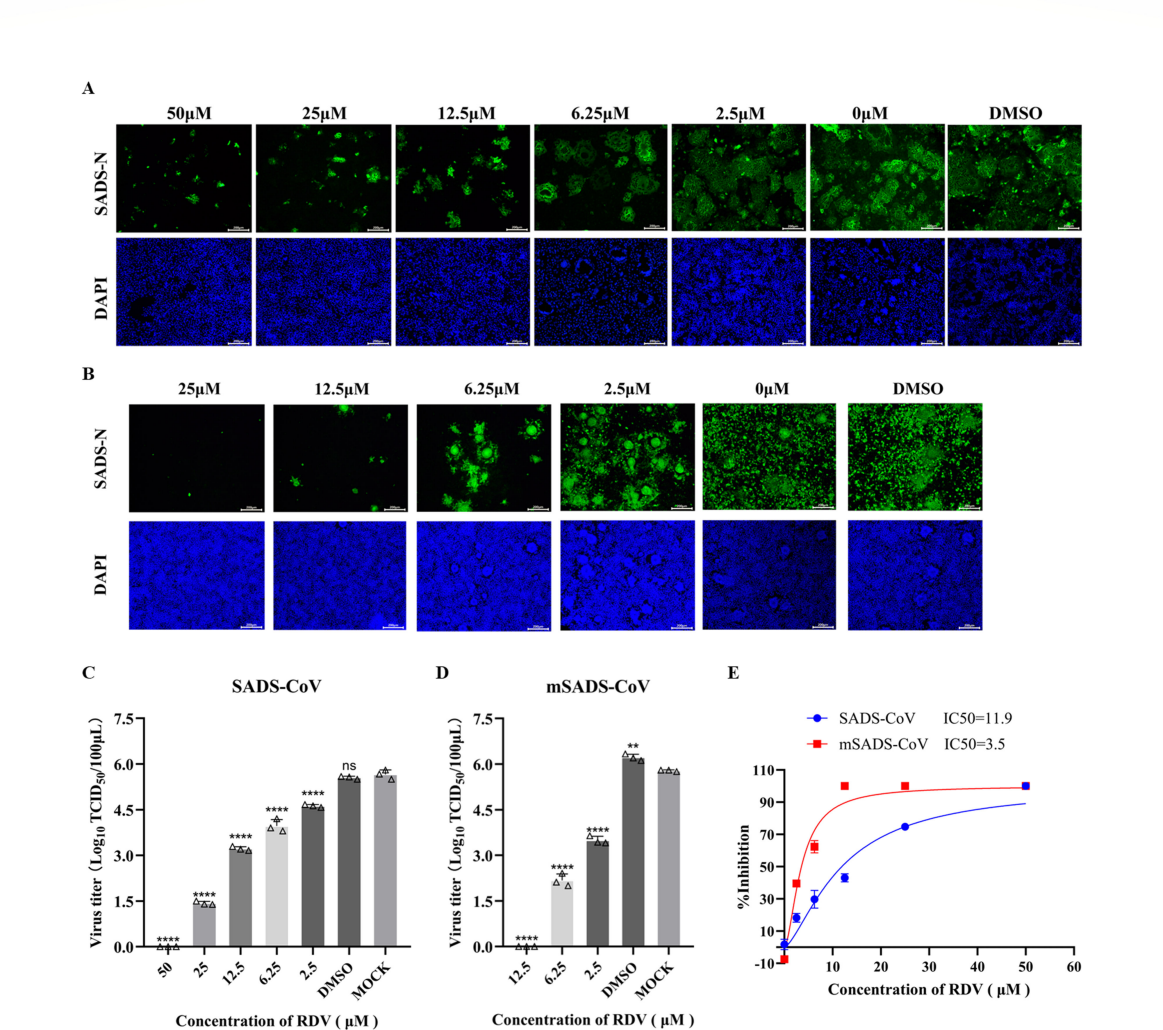
*In vitro* validation of RDV’s inhibitory efficacy against mSADS-CoV and SADS-CoV. (**A**) Determination of SADS-CoV infection in Vero cells treated with RDV at various concentrations by IFA staining. (**B**) Determination of mSADS-CoV infection in LR7 cells treated with RDV at various concentrations by IFA staining. (**C**) Viral titers of supernatants from SADS-CoV-infected Vero cells treated with different concentrations of RDV as expressed in log_10_ TCID_50_/100 μL. Each data point represents the mean of three technical replicates per sample. (**D**) Viral titers of supernatants from mSADS-CoV-infected LR7 cells treated with different concentrations of RDV. Each data point represents the mean of three technical replicates per sample. (**E**) The IC_50_ of RDV against mSADS-CoV in LR7 cells, as determined by infectious virus titer assay, was 3.5 μM. In contrast, the IC_50_ against SADS-CoV in Vero cells was 11.9 μM. No significant cytotoxicity was detected for either compound in both LR7 and Vero cells. Error bars represent the standard deviation. **, *P* < 0.01; ****, *P* < 0.0001; ns means not significant.

After 18 h of mSADS-CoV infection, cell samples were fixed using 4% polyformaldehyde for IFA staining, while the supernatants were collected for subsequent TCID_50_ assay. The same protocol was used to verify the *in vitro* inhibitory effect of RDV on SADS-CoV, now in Vero cells. For both viruses, the IFA analysis revealed that RDV can inhibit the replication of both SADS-CoV and mSADS-CoV in a dose-dependent manner (Fig. 4A and B). The titers of SADS-CoV and mSADS-CoV in the supernatants decreased as RDV concentration increased, with a highly significant difference observed between RDV-treated groups and untreated controls. (Fig. 4C and D). Based on the data presented in Fig. 4C and D, we calculated the half-maximal inhibitory concentration (IC_50_) of RDV against both viruses. As shown in Fig. 4E, the IC_50_ of RDV against mSADS-CoV replication in LR7 cells was 3.5 μM, whereas it was 11.9 μM against SADS-CoV in Vero cells. These results demonstrate that RDV effectively inhibits viral replication *in vitro*, supporting its use as a positive antiviral control for evaluating the suitability of the mouse infection model.

**Fig 5:**
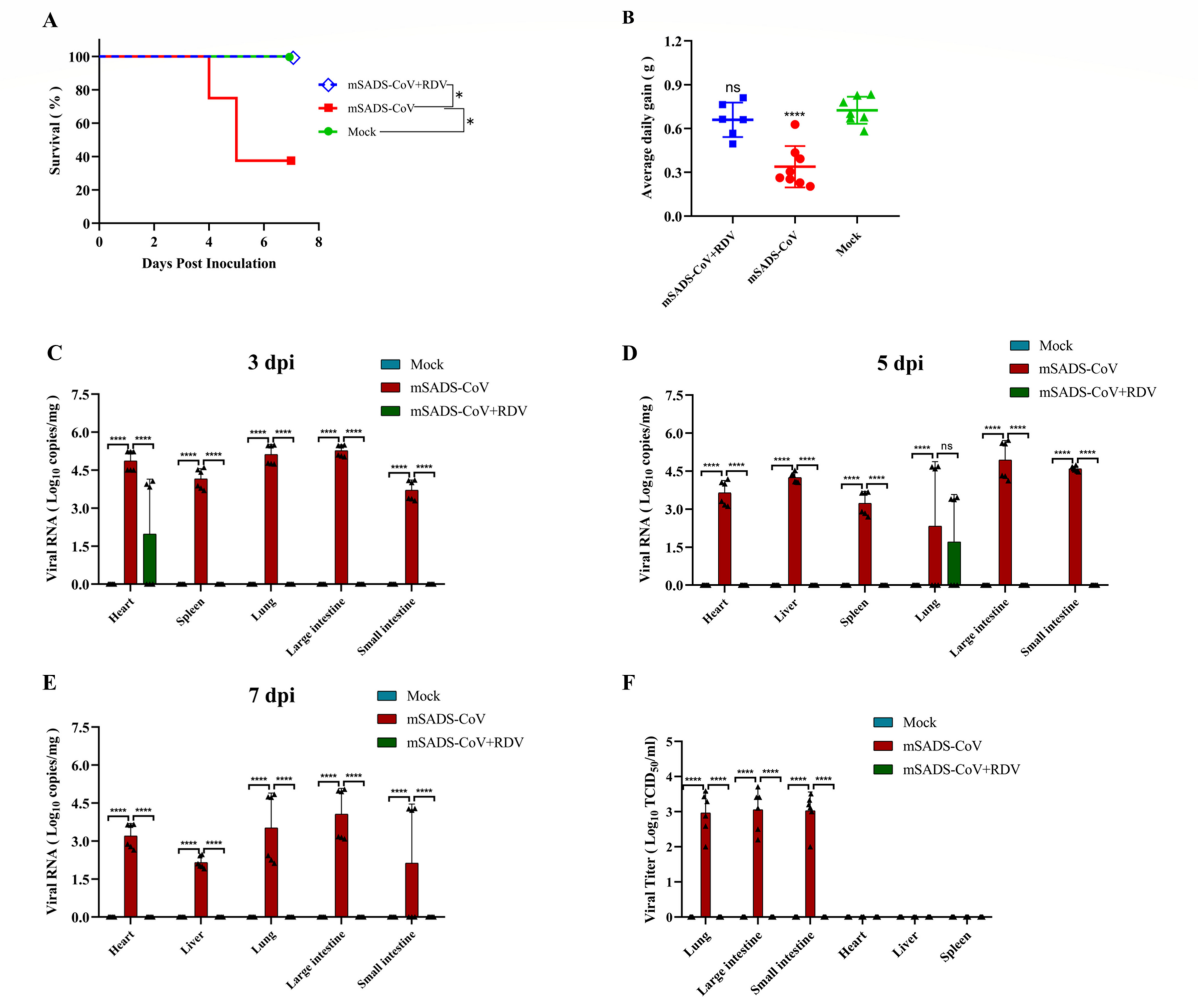
The effect of RDV on the infection by mSADS-CoV in 2-day-old BALB/c mice. (**A**) Survival curves of RDV-treated and untreated mSADS-CoV-infected mice and non-infected control animals. (**B**) Average daily weight gain of RDV-treated and untreated mSADS-CoV-infected mice and non-infected control animals. (**C–E**) Viral loads (RNA genome copies/mg) in the heart, liver, spleen, lung, large intestine, and small intestine of RDV-treated and untreated mSADS-CoV-infected mice and in non-infected control animals at (**C**) 3 dpi, (**D**) 5 dpi, and (**E**) 7 dpi. Each data point represents an individual technical replicate. (**F**) Viral loads (TCID_50_/mL) determined in cleared tissue homogenate supernatants across experimental groups. Each data point represents the mean of three technical replicates per sample. Error bars represent the standard deviation. ****, *P* < 0.0001; ns means not significant.

We subsequently studied the effect of the drug in animals. As observed by the clinical and weight monitoring, all mSADS-CoV + RDV and mock group mice survived, whereas in the mSADS-CoV group, mice started to die at 4 dpi, reaching 62.5% mortality by 7 dpi when the experiment ended (Fig. 5A). While the animals in the mSADS-CoV + RDV group grew at the same pace as those in the mock group, a severe growth retardation was observed in the untreated, infected mice as judged by the significantly lower average daily weight gain of this group (Fig. 5B).

Tissue samples collected from mice at 3, 5, and 7 dpi were subjected to qRT-PCR to assess the impact of RDV on the viral loads in the tissues of the mSADS-CoV-infected mice. In the mSADS-CoV + RDV group, viral genomic RNA was hardly detectable; only the hearts at 3 dpi and the lungs at 5 dpi scored positive (Fig. 5C through E). Viral infectivity in the tissues was quantitated by TCID_50_ determination, inoculating serial dilutions of cleared tissue homogenates onto LR7 cells. Again, an infectious virus was observed in the non-treated mice, not in RDV-treated animals (Fig. 5F). Remarkably, while high viral RNA loads were detected by qRT-PCR in all tissues tested, only lung, small intestine, and large intestine were positive in the infectivity assay. We have no explanation for why the heart, liver, and spleen came out negative, but we assume this had a technical cause, perhaps related to virus inactivation, for instance, due to the homogenization of small tissue fragments (heart and spleen) or due to cytotoxicity of the homogenate on LR7 cells (liver).

**Fig 6:**
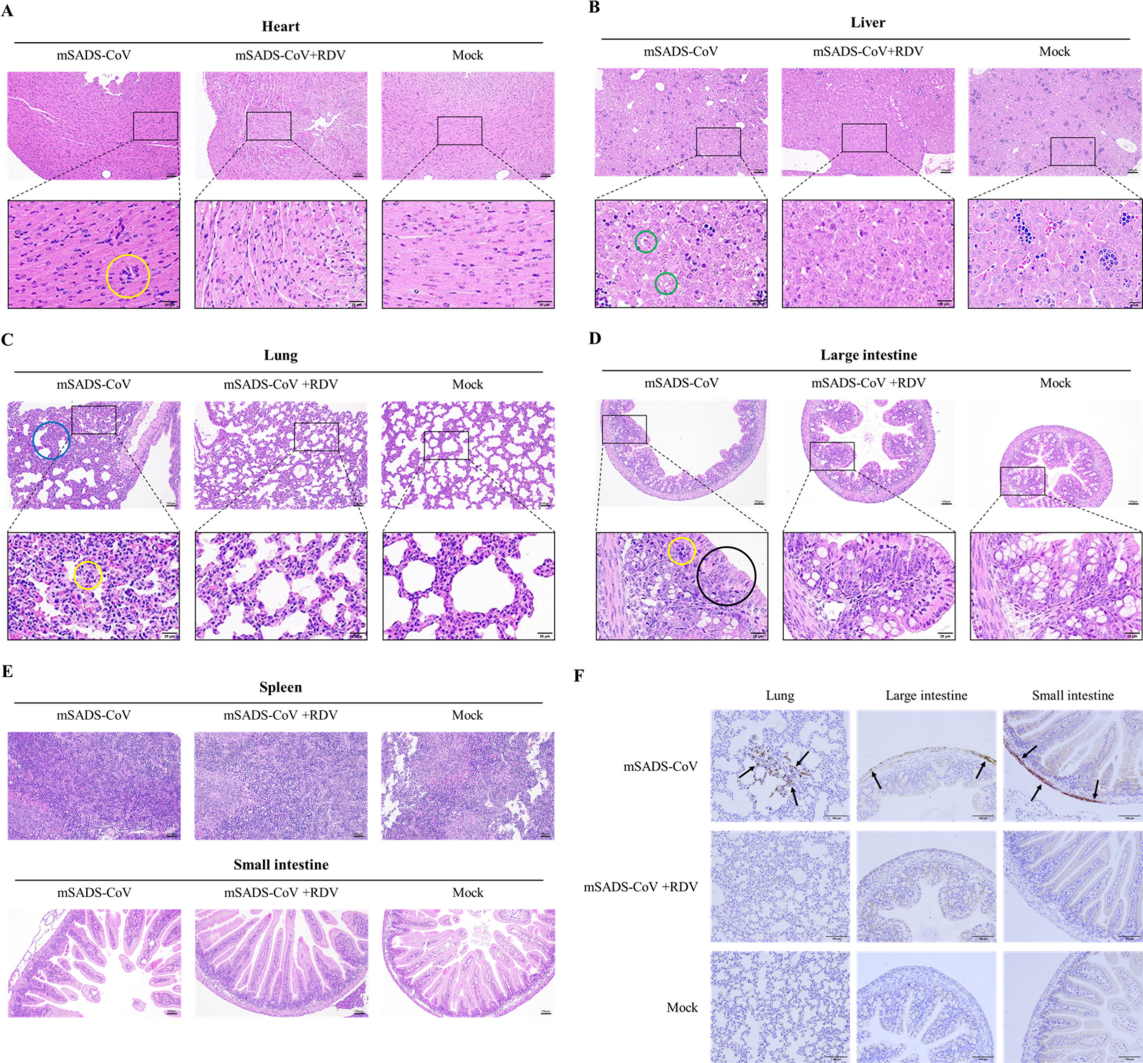
Pathological and immunohistochemical analysis of 2-day-old BALB/c mice infected with mSADS-CoV. (**A–E**) Hematoxylin and eosin (H&E) staining of different tissue sections from animals from the mSADS-CoV group, the mSADS-CoV + RDV group, and the mock group at 5 dpi. Yellow circles denote inflammatory cell infiltration; green circles denote polymorphic cytoplasmic lipid droplets; blue circles denote concurrent alveolar epithelial hyperplasia and septal thickening; and black circles denote marked reduction of intestinal goblet cells. (**F**) Immunohistochemical analysis of different tissue sections from animals from the mSADS-CoV group, the mSADS-CoV + RDV group, and the mock group at 5 dpi. Black arrows indicate mSADS-CoV antigen distributed in the alveolar walls of the lungs and the basal layers of the large and small intestines (representative positive signals are indicated).

Histopathological analysis of tissue samples from the mSADS-CoV group taken at 5 dpi revealed distinct pathological alterations across multiple organs (Fig. 6A through E): mild inflammatory cell infiltration was observed in cardiac tissue; hepatocytes exhibited variably sized lipid droplets; the splenic red pulp displayed marked expansion; pulmonary tissues exhibited alveolar epithelial hyperplasia, thickening of alveolar septa, and extensive inflammatory cell infiltration within the lung interstitium; and the large intestine presented a reduction in goblet cell numbers and disorganized epithelial cell architecture, accompanied by inflammatory cell infiltration in the mucosal layer. Of note, no significant pathological changes were detected in the small intestine. The mSADS-CoV + RDV group exhibited no notable histopathological alterations and was comparable to the control group. Immunohistochemical analysis further corroborated the efficacy of RDV in inhibiting mSADS-CoV infection in mice. Using a mouse monoclonal antibody targeting the SADS-CoV N protein, positive staining was observed in the alveolar walls of the lungs and the basal layers of the large and small intestines in the mSADS-CoV group. In contrast, no clear staining was observed in the mSADS-CoV + RDV group, as in the mock group (Fig. 6F).

## DISCUSSION

Scientific evidence has convincingly demonstrated the distinctive identity of SADS-CoV compared to other prevalent porcine enteric pathogens such as porcine epidemic diarrhea virus (PEDV), porcine deltacoronavirus (PDCoV), and transmissible gastroenteritis virus (18, 19). Immunologically, this distinction directly correlates with the lack of cross-protective efficacy of existing commercial vaccines developed for these related viral pathogens against SADS-CoV infection. Epidemiological evidence suggests SADS-CoV to have emerged from bats into rodents by cross-species transmission before spilling over into porcine hosts, its establishment in domestic swine populations creating substantial zoonotic risks through amplified human exposure opportunities (20, 21). This transmission paradigm raises significant concerns regarding potential anthroponotic spread. Elucidating the pathogenic mechanisms underlying SADS-CoV infection therefore represents a critical research priority for public health security. Concurrent advancements in developing prophylactic and therapeutic countermeasures are urgently required to mitigate pandemic risks. The establishment of robust, reproducible animal infection models constitutes a fundamental prerequisite for investigating viral pathogenesis and for enabling the pre-evaluation of drug and vaccine candidates.

In order to improve or create a mouse infection model, one of the currently emerging approaches is by generating transgenic animals made (more) susceptible by the introduction of the cell entry receptor for the particular virus. Well-known examples in the field of coronaviruses are the mouse strains that express the human receptor for SARS and MERS coronaviruses, ACE2 (22, 23) and DPP4 (24), respectively. This approach obviously requires the relevant receptor to be known, which is not the case for SADS-CoV. As an alternative, rather than adapting the mouse to the virus, an inverse approach is feasible as well. In its classical format, this may be achieved by passaging the virus serially through mice, thereby enabling the virus to evolutionary adapt to the murine host. A more directed approach to achieve such adaptation involves the retargeting of the virus by genetic engineering. The ability to do so was originally demonstrated some 25 years ago by replacing the spike protein’s ectodomain from one coronavirus by that of another; the resulting chimeric virus adopted the tropism of the latter. Thus, MHV was retargeted to feline cells using a feline coronavirus S sequence (17); the feline coronavirus was inversely retargeted to mouse cells (25) and an avian coronavirus similarly to mouse cells (26).

A convenient way to generate such chimeric coronaviruses is by targeted RNA recombination technology (16). As also demonstrated in the present study, this method enables the selection of the recombinant virus simply by growth on cells of the intended target species, in our case mouse cells. As MHV naturally infects mouse hosts, exhibiting high infectivity and pathogenicity characterized by hepatitis, enteritis, thymic atrophy, and induction of autoimmune responses (27, 28), the chimeric mSADS-CoV generated in this study theoretically possesses enhanced mouse infectivity compared to wild-type SADS-CoV, a hypothesis substantiated by our findings. The successful application of S protein ectodomain replacement in rescuing multiple coronaviruses (17, 25, 26, 29, 30) establishes its utility as a versatile platform for coronavirus research. This methodology can be effectively employed to generate chimeric recombinants of diverse coronaviruses, particularly facilitating the development of mouse-adapted viral variants. Such advancements provide a novel methodological framework for establishing mouse infection models across various coronavirus species, thereby enhancing our capacity to study coronavirus pathogenesis and host adaptation mechanisms.

Our observations demonstrated that SADS-CoV caused subclinical infection in neonatal BALB/c mice, exhibiting limited replicative capacity in 2-day-old, less in 7-day-old animals, followed by rapid viral clearance. These observations differ from those reported by Chen et al. with respect to the clinical impact of the infection (11). While similarly observing the age dependence of infection, the inoculations with SADS-CoV in their studies were lethal in the 2-day-olds, lethality decreasing in 5- and further in 7-day-old animals. A possible explanation for the difference might be the inoculation route, which was intragastrical in their studies and intraperitoneal in ours.

Chimeric mSADS-CoV exhibited subclinical infection in 7-day-old BALB/c mice, characterized by transient, low-level viral replication. However, in 2-day-old BALB/c mice, the virus induced lethal infection, accompanied by significant growth retardation, elevated tissue viral titers, and mortality rates exceeding 60%. Post-mortem examination revealed intestinal pathology characterized by wall edema, thinning, and the presence of foamy luminal contents, consistent with the SADS-CoV-induced phenomena observed in piglets and in mouse models upon intracerebral infections (12, 31). Although we have no data regarding the actual tropism of mSARS-CoV (nor of SADS-CoV) in the mice, the pathological changes caused by mSADS-CoV infection in 2-day-old BALB/c mice are very similar to those caused by SADS-CoV infection in porcine, the neonatal mouse model thus appearing to provide a convenient experimental model for studying SADS-CoV pathogenesis in swine.

RDV, a broad-spectrum antiviral nucleotide analog, exerts its pharmacological effect through premature termination of viral RNA synthesis via inhibition of viral RdRp activity (32). *In vitro* studies have established RDV’s inhibitory efficacy against multiple RNA viruses, encompassing SARS-CoV-2, SARS-CoV, MERS-CoV, Ebola virus, respiratory syncytial virus, Nipah virus, PEDV, PDCoV, and SADS-CoV (33–37). Therefore, RDV was selected in this study to evaluate the suitability of the mSADS-CoV mouse infection model for assessing the efficacy of antiviral drugs targeting viral replication. Unlike the non-treated mSADS-CoV-infected animals, most of which died, mice treated with RDV exhibited normal weight gain, no mortality, and a marked decrease in tissue viral load. Pathological lesions induced by viral infection were effectively alleviated, indicating that RDV potently inhibits mSADS-CoV replication *in vivo*, attenuates viral proliferation and infection across multiple tissues and organs, and consequently demonstrates significant antiviral activity. The observations demonstrate that the experimental platform can be used for antiviral studies targeting the viral replication machinery.

Histopathological analysis of 2-day-old mSADS-CoV-infected mice at 5 dpi revealed extensive viral replication in multiple organs, including pulmonary, intestinal, and splenic tissues, resulting in severe pathological alterations. The observed pulmonary and intestinal lesions exhibited remarkable similarity to those induced by SADS-CoV in neonatal mice (11). Our immunohistochemical analysis demonstrated distinct tissue tropism patterns: anti-SADS-CoV N protein staining of mSADS-CoV-infected mouse tissues was predominantly localized in alveolar walls and intestinal basal layers. In general agreement herewith, upon intragastrical administration of SADS-CoV to 2-day-old mice, viral antigen was found in alveolar walls, small intestinal villous epithelial cells, and large intestinal basal layers (11), while intracerebral SADS-CoV administration to 7-day-old mice gave rise to viral antigen appearance in alveolar walls and intestinal epithelial cells (12). These findings suggest that the spike gene modification of SADS-CoV that created the chimeric mSADS-CoV did not dramatically affect the overall tropism features of the virus in neonatal mice.

In summary, the SADS-CoV mouse model presented in this study provides a valuable tool for investigating the molecular mechanisms of SADS-CoV replication, in particular for the screening of antiviral drugs targeting viral replicase genes and for validating host factors involved in coronavirus replication. It also offers new insights for developing mouse infection models for other coronaviruses. These models have, however, limitations, which relate to the replacement of the spike protein’s ectodomain and which may hence affect viral tropism and cell entry features, as well as molecular processes underlying disease development. As a consequence, the models are not suitable for the study of entry inhibitors or for the evaluation of S protein targeting antibodies or vaccines.

## MATERIALS AND METHODS

### Biosafety statement

All work involving viruses was conducted in strict accordance with the national regulations on biosafety management of pathogenic microorganisms. Experiments with MHV-A59, SADS-CoV, and mSADS-CoV were performed in a biosafety level 2 facility.

### Cell lines, viruses, and antibodies

African green monkey kidney Vero cells (ATCC CCL-81) were purchased from the American Type Culture Collection (ATCC), and mouse LR7 cells were kindly provided by Professor Chunhua Li from the Shanghai Academy of Agricultural Sciences. The SADS-CoV GDS04 strain (GenBank accession number: MF167434.1) was kindly provided by Professor Yongchang Cao from Sun Yat-sen University; the MHV-A59 strain was purchased from the ATCC. The anti-SADS-CoV N protein monoclonal antibody, anti-SADS-CoV S protein monoclonal antibody, and monoclonal antibody targeting the MHV-S protein used in this study were prepared and preserved in our laboratory.

### Plasmid construction

Viral genomic RNA was isolated from the supernatants of SADS-CoV-infected Vero and MHV-A59-infected LR7 cells via TRIzol (Invitrogen). The extracted RNA was immediately used for cDNA synthesis according to the manufacturer’s instructions. Using the cDNA of these two viruses as templates, we amplified, cloned, and inserted the required fragments into the pUC57 vector via 2× MultiF Seamless Assembly Mix (ABclonal) according to the manufacturer’s instructions (amplification primers are listed in Table 1). The resulting recombinant plasmids pUC57-SADS-ORF1ab-Spike-3′UTR and pUC57-SADS-ORF1ab-MHV Spike-3′UTR were confirmed by Sanger sequencing to ensure their accuracy and fidelity.

**TABLE 1:** Primer sequence list for plasmid construction

Primer	Primer sequence (5′−3′)
SADS-T7-5UTR-F	TAATACGACTCACTATAGGACTTAAAGATATAATCTATC
SADS-5UTR-ORF1a-R	GGAATTCCTCCAAAACATAATTAGAATTAGAGG
SADS-ORF1b-F	GTTTTGGAGGAATTCCATTATGTCAGTGATGCTGA
SADS-ORF1b-3UTR-R	ACGGTGGCCTTAATTAATTTTTTTTTTTTTTTTTTTTGTGTATCA
pUC57-vector-F	ATTAATTAAGGCCACCGTGGCCAAGGGC
pUC57-vector-R	CTATAGTGAGTCGTATTAGGATCCCCGGGGGGATCCGATATCTAG
MHV-Spike-F	ATGCTGTTCGTGTTTATTCT
MHV-Spike-R	CCAAGGCCATTTCACATACA
pUC57-SADS-VF	TGTGAAATGGCCTTGGTGGCAGTGGCTGCTTATA
pUC57-SADS-VR	TAAACACGAACAGCATTCATTTAGTTGTTACAAGATGGTTGCT

### Rescue of the recombinant virus

The pUC57-SADS-ORF1ab-MHV Spike-3′UTR was linearized by PacI digestion and transcribed *in vitro* using an RNA transcription kit from Ambion following the manufacturer’s instructions. The resulting RNA transcripts were immediately placed on ice for electroporation. Vero cells that had been infected with SADS-CoV 5 h earlier were trypsinized and washed twice with PBS. The SADS-CoV-infected Vero cells were resuspended in PBS, and the suspension was transferred to an electroporation cuvette. The *in vitro* transcribed RNA was gently admixed, and the mixture was quickly placed in a Gene Pulser X cell electroporation system (Bio-Rad) for electroporation: a 450 V, 50 μF pulse was applied three times. The cells were quickly resuspended in cell growth medium and evenly plated onto a monolayer of LR7 cells. The cells were cultured overnight at 37°C with 5% CO_2_ and washed with PBS, and the medium was replaced by maintenance medium (DMEM with 2% fetal bovine serum) for continued culture, with regular observation for cytopathic effects.

### Plaque assay

The plaque overlay medium was prepared in advance by mixing phenol red-free 2× MEM with 1.8% low-melting-point agarose at a 1:1 ratio. The virus was serially diluted 10-fold in viral culture medium and inoculated onto cells (in 12-well plates) that had reached an appropriate confluence. After a 2-h incubation, the inoculum was removed, and an appropriate amount of the overlay medium was added to each well. Plates were further incubated for 3 days, followed by fixation with 4% paraformaldehyde at 37°C for 15 minutes. The overlay was then removed, and plaques were stained with 1% crystal violet for 1 h before observation. Viral titers were calculated and expressed as PFU per milliliter. SADS-CoV was propagated in Vero cells using DMEM supplemented with 10 μg/mL trypsin as the viral culture medium, while mSADS-CoV was cultured in LR7 cells with DMEM containing 2% fetal bovine serum as the viral culture medium.

### TCID_50_

The virus stock to be tested was serially diluted 10-fold in viral culture medium. Supernatants from animal serum and tissue homogenates were filtered through a 0.22 μm sterilizing filter prior to 10-fold serial dilution. Each dilution was inoculated onto cells (in 96-well plates) at 100 μL per well, with eight replicate wells per dilution. Observation began 72 h post-inoculation, and the number of wells showing a CPE was recorded daily until no new CPE appeared, typically around 96 h. The assay was performed in triplicate. The TCID_50_ value of the tested virus was calculated using the Reed–Muench method.

### Mouse infection experiments

Three-week-old SPF-grade BALB/c and C57BL/6 mice were randomly divided into experimental (*n* = 10) and control groups (*n* = 5) and inoculated with mSADS-CoV by intraperitoneal injection. Each mouse in the experimental group was injected with 1 mL of mSADS-CoV virus solution at a titer of 7 × 10^6.2^ PFU/mL, while the control group mice were injected with an equal volume of saline. We infected animals using the highest titer of mSADS-CoV produced from *in vitro* cell culture. Two-day-old and seven-day-old SPF-grade BALB/c mice (*n* = 6) were inoculated by intraperitoneal injection with 20 µL/g of an mSADS-CoV or SADS-CoV virus solution at a titer of 7 × 10^6.2^ PFU/mL; control animals (*n* = 3) received 20 µL/g of saline.

### Evaluation of the *in vivo* inhibitory effect of RDV on viral infection in mice

Two-day-old SPF-grade BALB/c mice were randomly divided into three groups (*n* = 8), one virus infection group (mSADS-CoV group), one RDV-treated infection group (mSADS-CoV + RDV group), and one control group (mock group). Due to the dependency of 2-day-old mice on maternal care during lactation, experimental groups were maintained in separate litters, with group sizes (five to eight pups) determined by natural birth rates. The mice in the two virus infection groups were inoculated by intraperitoneal injection with 20 µL/g of mSADS-CoV at a titer of 7 × 10^6.2^ PFU/mL. In parallel, mice in the mock group similarly received saline. The mice in the mSADS-CoV + RDV group were administered RDV by subcutaneous injection of a dose of 25 mg/kg/day (drug solvent formulation: 2% DMSO, 40% PEG300, 5% Tween 80, and 53% saline), with the first administration at −1 dpi and subsequent additional administrations every 24 h; these mice were designated the mSADS-CoV + RDV group. The animals in the mSADS-CoV group received subcutaneous injections with the same volume of drug solvent without the drug at the times when the mSADS-CoV + RDV group received the drug. At the same time, the animals in the mock group similarly received injections with the same volume of drug solvent.

### Evaluation of viral loads in tissues by quantitative reverse transcription PCR

All collected mouse tissue samples were weighed, homogenized in PBS, and subjected to total RNA extraction. After RNA was extracted from tissue samples, reverse transcription was performed according to the instructions of the Takara reverse transcription kit (RR036A). The primers used were designed on the basis of the conserved sequences of the SADS-CoV N gene and the MHV-A59 S gene (Table 2), and reverse transcription PCR was performed using DNA polymerase (CWBIO, China). The primers for quantitative fluorescence detection were designed on the basis of the conserved region of the SADS-CoV N gene, with the following primer sequences: forward primer SADS-CoV-qPCR-F: GTGCTAAAACTAGCCCCACAG; reverse primer SADS-CoV-qPCR-R: TGATTGCGAGAACGAGACTGTG. Fluorescence quantitative detection was performed via the Vazyme AceQ Universal SYBR qPCR Master Mix on the CFX96 and CFX384 instruments from Bio-Rad.

**TABLE 2:** Primer sequence list for RT-PCR

Primer	Primer sequence (5′−3′)
MHV-S-F	GACTTTGCATTTCTGGTGATAGAGGA
MHV-S-R	CAAGGCCATTTCACATACATTTCATATG
SADS-CoV-N-F	CACAGGTCTTGGTGTTCGCAATCG
SADS-CoV-N-R	ACCGTGCTGAACGAGGTCACT

### Detection of infectious virus in tissues of infected mice

To determine whether infectious virus was present in 2-day-old BALB/c mice infected with mSADS-CoV, positive tissue samples were homogenized and centrifuged to obtain tissue homogenate supernatants. The supernatants were filtered and inoculated onto LR7 cells, which were then monitored for the appearance of CPEs. Cells displaying CPE, along with their supernatant, were subjected to freeze–thaw cycling and subsequent centrifugation. The resulting material was used to inoculate fresh LR7 cells. At 20 h post-inoculation, the cells were fixed and analyzed by IFA using an antibody against the SADS-CoV N protein to confirm viral identity.

### Evaluation of viral loads in tissues by infectivity titration

Mouse tissue samples collected at 3, 5, and 7 dpi were weighed and homogenized. The homogenates were then centrifuged to obtain the supernatants. These supernatants were then clarified by centrifugation and filtration and serially diluted in 10-fold steps, and each dilution was inoculated onto LR7 cell monolayers in 96-well plates. The plates were monitored for the development of CPE. The TCID_50_ values for each homogenate were calculated based on the CPE observations using the Reed–Muench method.

### Statistical analysis

Statistical analyses were performed via GraphPad PRISM 8.0.1. For comparisons between the two experimental groups, two-tailed Student’s *t*-tests were performed to determine significant differences. Statistical significance was as follows: *, *P* < 0.05; **, *P* < 0.01; ***, *P* < 0.001; ****, *P* < 0.0001; ns means not significant.
